# A Feature-Enhanced Network for Vegetable Disease Detection in Complex Environments

**DOI:** 10.3390/plants15081182

**Published:** 2026-04-11

**Authors:** Xuewei Wang, Jun Liu

**Affiliations:** Shandong Provincial University Laboratory for Protected Horticulture, Weifang University of Science and Technology, Weifang 262700, China; wangxuewei@wfust.edu.cn

**Keywords:** vegetable disease detection, deep learning, feature fusion, object detection

## Abstract

Accurate vegetable disease detection in complex cultivation environments remains challenging because early lesions are often small, low-contrast, and easily confounded by cluttered backgrounds. To address this issue, we propose VDD-Net, a feature-enhanced detection network based on YOLOv10 for robust vegetable disease detection in protected agriculture. The proposed framework integrates three modules: a receptive field enhancement (RFE) module to improve local perception of small lesions, an adaptive channel fusion (ACF) module to strengthen multi-scale feature aggregation and suppress background interference, and a global context attention (GCA) module to capture long-range dependencies and improve contextual discrimination. Experiments on a custom vegetable disease dataset showed that VDD-Net achieved an mAP@0.5 of 95.2% with only 7.78 M parameters. To further evaluate robustness, zero-shot cross-domain testing was conducted on the PlantDoc dataset, where VDD-Net achieved an mAP@0.5 of 76.5%, outperforming the baseline and showing improved generalization to natural scenes. In addition, after TensorRT optimization and FP16 quantization, the model maintained real-time inference on edge platforms, reaching 89.3 FPS on Jetson AGX Orin and 24.2 FPS on Jetson Nano. These results indicate that VDD-Net provides a practical balance among detection accuracy, cross-domain robustness, and deployment efficiency for intelligent disease monitoring in modern agriculture.

## 1. Introduction

Vegetable production is a key component of global agriculture and plays an essential role in food security and economic stability [1]. However, with the expansion of intensive cultivation and ongoing climate change, vegetable diseases have become increasingly frequent and severe, leading to substantial losses in yield and quality [2,3]. Recent studies have shown that plant diseases continue to impose major economic burdens worldwide and pose a persistent threat to the resilience of food supply systems [4]. In conventional practice, disease diagnosis still relies heavily on visual inspection by experts or experienced growers. This process is labor-intensive, time-consuming, and prone to subjective bias, often resulting in missed or incorrect diagnoses. As a result, it is not well suited to the demands of large-scale, real-time monitoring in smart agriculture [5]. Developing efficient, objective, and non-destructive disease detection methods has therefore become an important research priority.

In recent years, deep learning methods, especially convolutional neural networks and vision transformers, have significantly advanced plant disease detection [6,7]. Although these methods often perform well under controlled conditions, early-stage lesions in real production environments usually appear as tiny spots or subtle texture changes. Their detection becomes substantially more difficult under illumination variation, occlusion, and cluttered backgrounds, and model performance often degrades sharply when transferring from laboratory or greenhouse settings to more diverse field scenes [8]. In addition, noise contamination, image blur, and image-quality degradation in complex agricultural scenes may further weaken lesion boundaries and texture details, thereby increasing the uncertainty of downstream recognition. Existing studies have shown that CNN-based denoising and image enhancement methods can recover useful information from degraded images to a certain extent, which also indicates that visual input quality has a fundamental influence on robust disease recognition [9]. This gap between benchmark performance and practical deployment remains a central challenge in developing robust and deployable disease detection systems.

Previous studies have gradually moved from image-level classification toward object detection and, more recently, toward architectures specifically designed for complex agricultural environments. Early work mainly applied classical CNN-based classification models and demonstrated the effectiveness of deep visual features for plant disease recognition [10]. On this basis, some studies further emphasized the role of diseased-leaf region localization in the recognition pipeline, indicating that extracting more discriminative leaf or lesion regions before final classification can reduce background interference and improve cross-crop disease classification performance. The leaf-image localization method proposed by Kurmi and Gangwar further demonstrated the importance of local-region awareness in visual plant disease analysis [11]. Meanwhile, the continued application of deep convolutional networks to leaf-image-based disease recognition has also advanced visual analysis of plant diseases. Related studies have shown that deep CNN models can learn discriminative disease features from leaf images and achieve strong performance in multi-class crop disease recognition tasks, thus laying an important methodological foundation for the subsequent transition from classification to detection and from simple scenarios to complex environmental modeling [12].

To simultaneously identify lesion categories and locations, one-stage detectors, particularly the YOLO family, have been widely adopted because of their favorable trade-off between speed and accuracy. Recent improvements include enhanced multi-scale feature extraction [13], transformer-based modeling of long-range dependencies [14], and attention mechanisms that encourage the network to focus on lesion regions [15]. Recent studies have further explored lightweight detectors, localization-guided pipelines, and detection models incorporating transformer-based representation ability for plant disease analysis, and these approaches have improved performance on benchmark datasets.

Despite this progress, several challenges remain. First, early disease symptoms often appear as very small lesions with limited spatial support. After repeated downsampling, their discriminative features may be weakened or lost, which increases the risk of missed detection [16,17]. Although lightweight detectors can improve inference speed, they often do so at the expense of fine-grained feature preservation, which limits their ability to detect subtle early symptoms [18,19]. Second, existing feature fusion strategies are often insufficient for handling lesions of different sizes and developmental stages. Simple multi-scale aggregation may introduce semantic conflicts and reduce the model’s ability to detect both small isolated spots and larger connected lesions [20]. Third, greenhouse scenes are highly complex. Soil, mulch, weeds, shadows, and illumination fluctuations can all introduce severe background interference. Under edge-side computational constraints, many existing global modeling approaches struggle to achieve both robustness and real-time performance. In particular, limited global context modeling weakens the network’s ability to establish long-range relationships across spatial locations and channels, making it harder to suppress non-disease interference and improve fine-grained discrimination [21,22,23,24]. These limitations reduce the practical value of existing models for precision crop protection. Improving small-lesion perception and environmental robustness through targeted architectural design is therefore highly desirable. Recent work has also suggested that combining local detail enhancement with broader contextual reasoning is especially important for complex scene analysis [25,26]. This perspective is highly relevant to agricultural visual recognition and may help advance deployable disease detection under resource-constrained conditions [27,28]. However, in complex agricultural scenarios, it remains insufficiently solved how to jointly achieve high sensitivity to small lesions, strong suppression of background interference, robust cross-domain transferability, and efficient edge deployment.

To address these challenges, we propose the Vegetable Disease Detection Network (VDD-Net), which is built on YOLOv10 and specifically designed for high-accuracy, robust disease detection in complex vegetable production environments. The proposed network incorporates three core modules. The receptive field enhancement (RFE) module expands the effective local receptive field through multi-branch dilated convolutions, thereby improving the representation of small lesions. The adaptive channel fusion (ACF) module optimizes multi-scale feature aggregation through channel re-weighting and reduces semantic conflicts during fusion. The global context attention (GCA) module models broader contextual dependencies and suppresses background interference more effectively. Experiments on a custom vegetable disease dataset show that VDD-Net achieves superior detection accuracy while maintaining real-time inference, demonstrating its potential for intelligent monitoring and precision disease management in modern vegetable production.

## 2. Results

### 2.1. Comparative Experiments

Given the limited availability of open-source methods specifically designed for complex vegetable disease detection across multiple crops and disease categories, we compared VDD-Net with nine representative object detection models to evaluate its overall performance in small-lesion recognition and background suppression.

**Figure 1:**
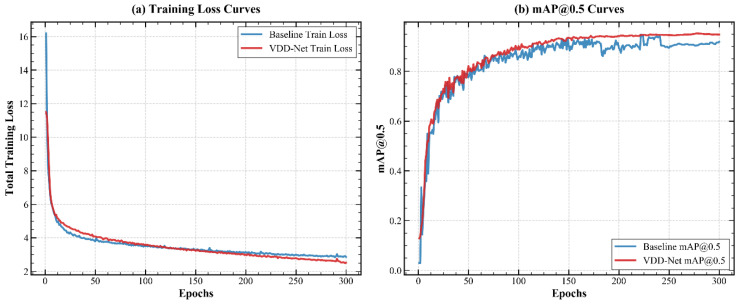
Comparison of convergence evolution between the proposed VDD-Net and the baseline YOLOv10s model. The training loss curves (**a**) and validation mAP@0.5 curves (**b**) demonstrate that VDD-Net achieves faster convergence and higher final detection accuracy.

The comparison methods covered three major categories: (1) the classical two-stage detector Faster R-CNN [29]; (2) lightweight YOLO-family models, including YOLOv8s [30], YOLOv9s [31], YOLOv10s [32], and YOLOv11s [33]; and (3) transformer-based end-to-end detectors, including DETR [34], Deformable DETR [35], Co-DETR [36], and RT-DETR [37]. For fairness, all models were retrained and evaluated using the same hardware platform and hyperparameter settings. The quantitative results are summarized in Table 1. The convergence behavior of the proposed model and the baseline during training is shown in Figure 1.

As shown in Table 1, VDD-Net achieved the best overall performance among all evaluated models, with an mAP@0.5 of 95.2% and an mAP@0.5:0.95 of 76.4% for the 30-category vegetable disease detection task. Compared with the baseline YOLOv10s, VDD-Net improved mAP@0.5 and mAP@0.5:0.95 by 4.1% and 8.2%, respectively. It also outperformed YOLOv11s by 2.7% in mAP@0.5. Among the transformer-based models, RT-DETR achieved strong accuracy, but VDD-Net still provided better performance with substantially fewer parameters. The proposed model contained only 7.78 M parameters, which was just 0.58 M more than YOLOv10s, while still maintaining 87.5 FPS.

**Figure 2:**
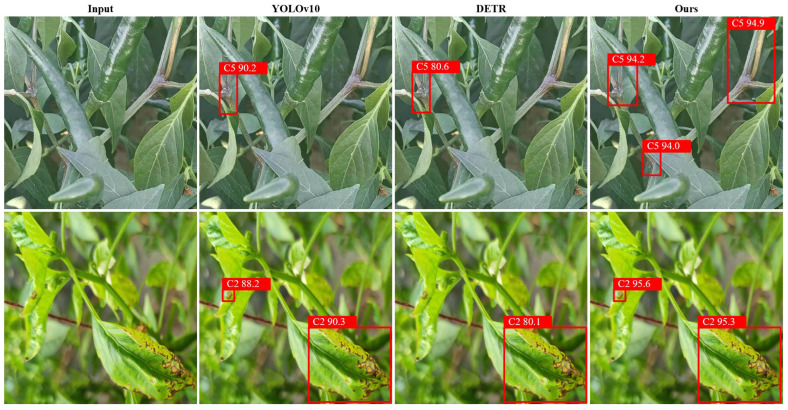
Comparison of vegetable disease detection results between the baseline YOLOv10s, DETR, and the proposed feature-enhanced VDD-Net. Red bounding boxes denote the predicted class and confidence score. VDD-Net effectively suppresses complex background interference (e.g., soil and healthy veins) and successfully identifies small, low-contrast early lesions that are missed by the baseline YOLOv10 and DETR models.

In representative cases such as early tomato blight and cucumber target spot, lesions often appear as tiny spots that can easily be confused with healthy veins, soil background, or specular reflections from water droplets. Conventional detectors such as Faster R-CNN and DETR are limited in these cases by insufficient preservation of fine spatial details and weak contextual modeling. By contrast, VDD-Net more effectively suppresses environmental noise while retaining discriminative lesion features, owing to the combination of multi-scale selective fusion and global contextual modeling. Representative qualitative comparison results are shown in Figure 2.

### 2.2. Ablation Experiments

To quantify the contribution of each proposed component, the RFE, ACF, and GCA modules were progressively added to YOLOv10s, and ablation experiments were conducted on PVDD. The results are presented in Table 2.

As shown in Table 2, each module contributed differently to the overall improvement. Adding the RFE module alone produced the largest gain in precision, increasing mAP@0.5 to 93.1%. This suggests that the multi-branch dilated convolutions help preserve lesion-edge texture and improve the discrimination of small disease regions from the background.

The ACF module produced a stronger improvement in recall. By selectively emphasizing informative channels during cross-scale fusion, it reduced the risk that small-target features would be overwhelmed by large-object or background features, thereby lowering the missed detection rate. Notably, this module introduced only 0.01 M additional parameters and had almost no effect on inference speed. The GCA module also improved performance, indicating that global contextual modeling helps suppress interference from non-disease regions. When all three modules were combined, the model reached an mAP@0.5 of 95.2%, confirming their complementary effects.

To further investigate the influence of different feature fusion mechanisms in the neck, we compared several channel fusion strategies after integrating the RFE and GCA modules. As shown in Table 3, all SCF-based variants outperformed the original BiFPN, confirming that selective channel weighting improves cross-scale aggregation by suppressing redundant background information and strengthening lesion-related responses. Among the evaluated strategies, SCF-2 achieved the highest mAP@0.5 of 95.2% while maintaining the parameter count at 7.78 M. Therefore, SCF-2 was selected as the final channel fusion strategy in the ACF module.

### 2.3. Cross-Dataset Generalization Validation

In practical agricultural applications, deep learning models often become dependent on the imaging characteristics of the training dataset and may perform poorly when transferred to new scenes. To evaluate the robustness of VDD-Net under domain shift, we used the public PlantDoc dataset as an external independent test set.

Because the output dimension of the detection head is determined by the training categories, this experiment focused on domain generalization rather than open-set recognition. Therefore, only PlantDoc categories overlapping with PVDD were retained to construct the external test subset. Under a zero-shot transfer setting, YOLOv10s and VDD-Net were trained only on PVDD and directly tested on the PlantDoc subset without any fine-tuning. The results are reported in Table 4.

As shown in Table 4, both models experienced performance degradation under cross-domain testing, which reflects the substantial distribution gap between controlled greenhouse images and more diverse real-world scenes. However, the degradation was smaller for VDD-Net. While YOLOv10s dropped to an mAP@0.5 of 68.3%, VDD-Net maintained 76.5%, indicating better cross-domain stability and stronger robustness to scene variation.

### 2.4. Edge-Side Deployment and Inference Testing

For deployment in agricultural equipment and Internet of Things nodes, a disease detection model must be both accurate and efficient under limited computational and energy resources. To evaluate the engineering feasibility of VDD-Net, deployment tests were conducted on two NVIDIA edge platforms: the higher-performance Jetson AGX Orin and the lightweight Jetson Nano.

During deployment, all tested models were optimized using TensorRT and FP16 quantization. Real-time video streams with batch size 1 were used for inference, and average latency, frames per second, and power consumption were recorded. The results are shown in Table 5.

On Jetson AGX Orin, VDD-Net introduced only a modest increase in computation and latency while maintaining 89.3 FPS and stable power consumption. On the more resource-constrained Jetson Nano, it still achieved 24.2 FPS, which indicates practical real-time capability. These results show that VDD-Net achieves a favorable balance among detection accuracy, speed, and deployment cost for edge-side agricultural applications.

## 3. Discussion

### 3.1. Interpretation of Performance Gains

The improved performance of VDD-Net can be attributed to the complementary effects of the RFE, ACF, and GCA modules. In protected cultivation environments, disease symptoms often appear as small lesions with weak contrast, irregular boundaries, and interference from occlusion or background clutter. Under these conditions, conventional feature extraction may fail to preserve subtle lesion details. The RFE module enhances multi-scale local perception by enlarging the effective receptive field, which is beneficial for capturing fine-grained symptom patterns. Meanwhile, the ACF module strengthens multi-scale feature fusion by adaptively emphasizing informative channels and suppressing less relevant responses. The GCA module further improves representation learning by incorporating broader contextual dependencies, which helps the network distinguish true lesions from visually similar background structures. Overall, the three modules jointly improve local detail perception, cross-scale feature fusion, and global contextual reasoning, which together explains the superior performance of VDD-Net in complex vegetable disease scenes.

From a plant-physiology perspective, the visible manifestation of disease symptoms is also shaped by host stress-response networks, in which hormone signaling plays an important role in regulating tissue development, coordinating defense responses, and mediating environmental adaptation [38]. At the same time, reactive species and redox regulation are deeply involved in plant defense and stress adaptation, which further increases the variability of disease phenotypes across different host states and environmental conditions [39]. Although the present study focuses on image-based detection rather than mechanistic pathology, this context helps explain why lesion phenotypes may vary substantially across environmental conditions and developmental stages.

### 3.2. Generalization and Deployment Implications

In addition to strong in-domain performance, VDD-Net also showed good cross-dataset robustness on PlantDoc, suggesting that the model is less dependent on greenhouse-specific backgrounds and better able to capture disease-related visual cues. It should be noted that the PlantDoc experiment in this study is better regarded as a zero-shot cross-domain validation under partial category overlap, rather than a full domain adaptation evaluation. Because no target-domain fine-tuning or dedicated domain generalization baselines were included, these results mainly indicate that the proposed architecture has promising transfer potential across scenes, while more rigorous adaptation-oriented comparisons remain for future work.

Nevertheless, the present evidence is more appropriately interpreted as strong robustness within heterogeneous protected-cultivation conditions and preliminary transferability to an external dataset, rather than full validation across open-field environments. Compared with greenhouse scenes, real field conditions typically involve stronger illumination fluctuations, more complex natural backgrounds, heavier canopy occlusion, and greater imaging-device variation. Therefore, further multi-site, multi-season, and multi-device field validation is still necessary before broad practical deployment. This is important for practical applications, since many plant disease detection models experience a clear performance drop when transferred to more diverse or less controlled environments. The edge deployment results further support the practical value of the proposed model. After TensorRT optimization and FP16 quantization, VDD-Net maintained real-time inference on both Jetson AGX Orin and Jetson Nano while preserving strong detection performance. These results indicate that the proposed architecture achieves a useful balance among accuracy, robustness, and deployability, which is essential for intelligent disease monitoring in modern agricultural systems.

### 3.3. Limitations and Future Perspectives

This study still has several limitations. First, the current framework is based on single-image analysis and does not explicitly model temporal information, which may limit its performance in continuous monitoring scenarios involving motion blur, viewpoint changes, or dynamic symptom progression. Temporal information can provide cross-frame consistency constraints and disease-progression cues, and may therefore improve detection stability in continuous monitoring scenarios, especially when lesion expansion, leaf motion, and transient occlusion coexist. Future work may integrate video modeling, temporal feature aggregation, or multi-frame decision mechanisms to further enhance robust recognition in complex agricultural environments.

Second, the model mainly relies on visual features, whereas disease occurrence in greenhouse production is often closely related to environmental factors such as temperature, humidity, and illumination. In practical greenhouse management, environmental variables may provide informative priors even before visually obvious symptoms appear. Therefore, multimodal fusion may improve early recognition ability and enhance the stability of discriminating ambiguous samples and complex scenes. Future work may combine temperature, humidity, leaf-surface wetness, and illumination sensing with image features to build a more comprehensive multi-source disease detection framework for smart agriculture.

Moreover, the current experiments were conducted under a closed-set setting in which the training and test categories were predefined. As a result, the present study does not directly evaluate the open-set recognition ability of VDD-Net for unseen disease categories or entirely new crop-disease combinations. Future work may therefore investigate unknown-class rejection, open-set detection, and incremental category expansion strategies to improve adaptability in complex real agricultural scenarios.

In addition, typical failure cases mainly arise in three scenarios, namely severe occlusion that breaks lesion boundary information, extremely early symptoms that occupy only a few pixels, and lesion appearances that are highly similar to veins, specular reflections, or cluttered background textures. Compared with the baseline model, VDD-Net alleviates these difficulties to a certain extent, but false positives and missed detections caused by extremely weak visual signals and high background similarity cannot yet be completely avoided.

Overall, the present study provides a solid basis for developing more robust and practical plant disease detection systems for smart agriculture.

## 4. Materials and Methods

### 4.1. Dataset

**Figure 3:**
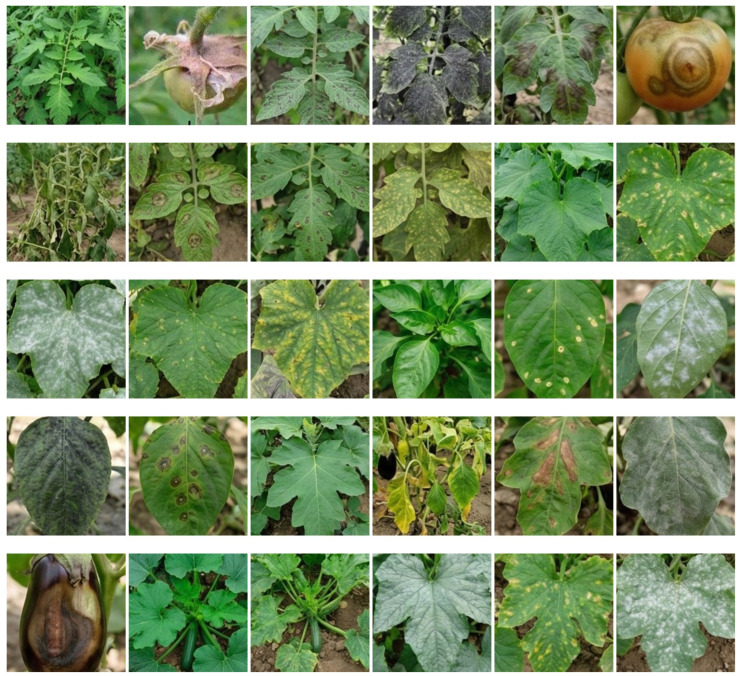
Example images from the PVDD. The dataset contains healthy and diseased categories from multiple vegetable crops under complex greenhouse conditions.

This study used the previously constructed Protected Vegetable Disease Dataset (PVDD) for model training and evaluation. The dataset captures disease characteristics under complex greenhouse conditions, including illumination changes, leaf overlap, and background interference, and covers 30 healthy and diseased categories across five crops: tomato, cucumber, pepper, eggplant, and zucchini. Disease instances were annotated using LabelImg, and the annotation information was stored in TXT files. A total of 30,000 manually annotated valid images were used in the experiments. Examples from the PVDD are shown in Figure 3.

To reduce the potential bias associated with a single data split and to improve evaluation reliability, a stratified five-fold cross-validation strategy was adopted. In each split, the dataset contained 24,000 images for training, 3000 for validation, and 3000 for testing.

### 4.2. Experimental Settings and Evaluation Metrics

VDD-Net was implemented in PyTorch (version 1.13.1), and all training and inference experiments were conducted on a workstation equipped with an NVIDIA GeForce RTX 4090 GPU with 24 GB of VRAM. During training, the stochastic gradient descent optimizer was used for parameter updates. A linear warm-up strategy was applied during the first three epochs to stabilize optimization, with an initial learning rate of 0.01 followed by cosine annealing decay. The momentum was set to 0.937, the weight decay coefficient to 0.0005, the batch size to 32, and the total number of training epochs to 300. Mosaic augmentation was disabled during the final 10 epochs so that the model could better adapt to the true data distribution.

Detection performance was evaluated using the standard object detection metrics precision, recall, mAP@0.5, and mAP@0.5:0.95. In addition, the number of parameters and frames per second were used to assess model complexity and inference efficiency. Because this study is primarily deployment-oriented, the number of parameters and FPS directly reflect memory footprint and practical inference throughput, and were therefore adopted as direct indicators of model complexity and deployability.

### 4.3. Overall Framework

As one of the latest YOLO variants, YOLOv10 preserves the high speed of one-stage detectors while reducing computational latency by eliminating non-maximum suppression. Based on this architecture, we constructed VDD-Net.

**Figure 4:**
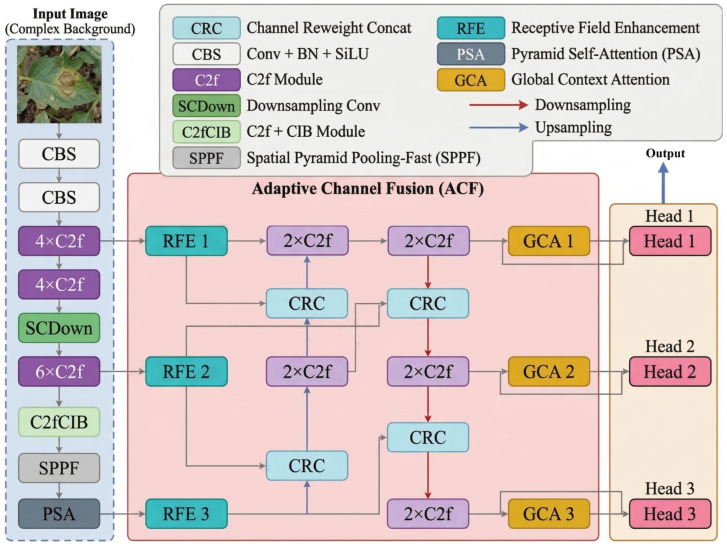
The overall architecture of the proposed VDD-Net. Built upon the YOLOv10 baseline, the framework integrates the Receptive Field Enhancement (RFE), Adaptive Channel Fusion (ACF), and Global Context Attention (GCA) modules to optimize multi-scale feature extraction and environmental robustness.

VDD-Net retains the original four-stage convolutional downsampling structure of the backbone and introduces three additional components: RFE, ACF, and GCA. The RFE module expands the receptive field of the backbone by means of a multi-branch dilated convolution design, thereby enhancing the representation of small-lesion regions. The ACF module improves the fusion pathway in the feature pyramid through channel re-weighting, strengthening multi-scale integration without introducing excessive computational cost. The GCA module models global contextual dependencies across channels and spatial locations, which helps improve the semantic association between small lesions and the surrounding scene. The overall architecture of VDD-Net is shown in Figure 4.

### 4.4. RFE Module

In dense greenhouse canopies, early disease symptoms often appear as tiny lesions with very limited pixel coverage. These subtle disease regions are easily overwhelmed by complex backgrounds such as soil, mulch, and weeds, which can lead to missed detections and false alarms. Although deep backbones provide increasingly abstract semantics, repeated downsampling also weakens the spatial detail needed to represent small targets. In addition, the limited receptive field of standard convolutions restricts the network’s ability to capture the local context surrounding lesions. To address these issues, we designed the RFE module with a lightweight multi-branch structure that combines multi-scale feature extraction with dilated convolutions.

**Figure 5:**
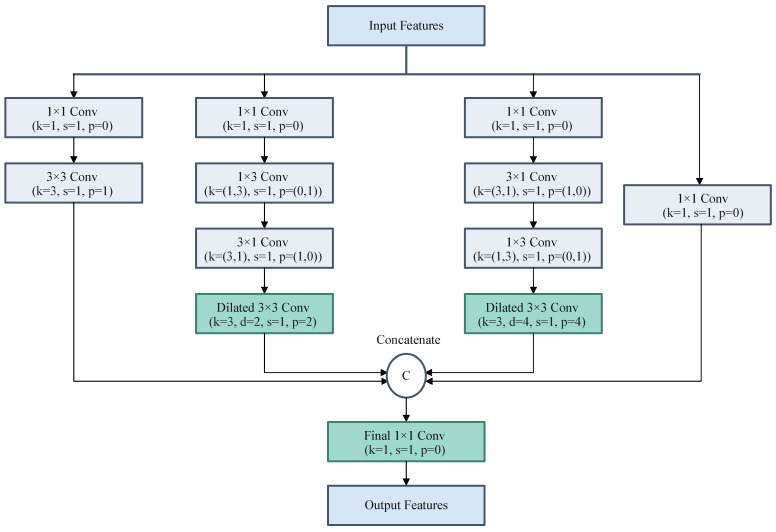
Detailed structure of the Receptive Field Enhancement (RFE) module. Multi-branch dilated convolutions are utilized to expand the effective receptive field while retaining fine spatial details for small-lesion representation.

The structure of the RFE module is shown in Figure 5. In the RFE design, each branch first applies a 1 × 1 convolution to adjust the channel dimension. One branch adopts a residual path to preserve key lesion information, whereas the other branches perform cascaded convolutions with kernel sizes of 1 × 3, 3 × 1, and 3 × 3. Dilated convolutions are added to the intermediate branches to enlarge the effective receptive field while preserving spatial resolution. In this way, the RFE module captures richer local contextual information and improves the representation of small-lesion targets.

Let the input feature map be $X\in R^{C\times H\times W}$. The output of the RFE module is formulated as

(1) $$X_{RFE}=\mathrm{Concat}(B_{0}(X),\;B_{1}(X),\;B_{2}\left(X),\;B_{3}(X)\right)$$

where $B_{0}\;$ denotes the residual-preserving branch, and $B_{1}$, $B_{2}$, and $B_{3}$ denote parallel branches with different convolutional combinations and dilation settings. According to Equation (1), the RFE module aggregates local features and multi-scale contextual cues while preserving spatial resolution, thereby improving the representation of tiny lesion regions.

### 4.5. ACF Module

In vegetable disease detection, shallow feature maps retain fine spatial details such as lesion edges and textures, whereas deeper feature maps contain richer semantic information. Effective cross-scale feature fusion is therefore essential for preserving small-lesion information. Based on the efficient topology of BiFPN, we propose the ACF module. Unlike the original BiFPN, which assigns scalar weights uniformly during feature aggregation, ACF introduces a selective channel fusion mechanism that adaptively recalibrates the importance of different channels across scales.

**Figure 6:**
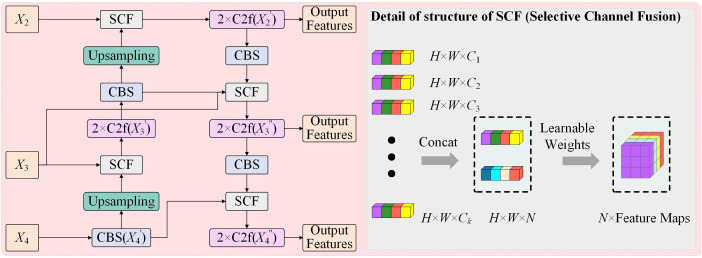
Detailed structure of the Adaptive Channel Fusion (ACF) module. This component optimizes top-down and bottom-up cross-scale feature aggregation by adaptively recalibrating channel weights.

The ACF module performs both top-down and bottom-up feature fusion. In the top-down path, higher-level feature maps are processed and upsampled before being fused with lower-level features using the selective channel fusion strategy. The bottom-up path mirrors this process, except that downsampling is performed by stride-2 convolutions. The resulting fused feature maps allow both low-level details and high-level semantics to be integrated more effectively. The structure of the ACF module is shown in Figure 6.

To optimize channel fusion, three channel re-weighting strategies were examined. Strategy 1 incorporated classical attention mechanisms such as SENet [40] and ECANet [41]. Strategy 2 directly learned normalized global channel weights after feature concatenation. Strategy 3 performed hierarchical weighting within each feature map before applying global weighting across feature maps. Based on the ablation results, all three strategies improved performance, whereas Strategy 2 showed the best overall balance. It was therefore adopted in the final ACF design.

As defined in Equations (2) and (3), let ${\left\{F_{i}\right\}}_{i=1}^{N}$ denote the feature maps from different scales. The adaptive channel weights are generated by global aggregation of concatenated features:

(2) $$[\alpha_{1},\alpha_{2},\ldots ,\;\alpha_{N}]\;=\;\phi (\mathrm{GAP}(\mathrm{Concat}(F_{1},F_{2},\ldots ,\;F_{N})))$$

where ϕ(⋅) denotes a lightweight channel mapping function and $\alpha_{i}\;$ represents the channel-wise importance of the i-th scale. The fused output of the ACF module is then expressed as

(3) $$\hat{F}=\sum_{i=1}^{N}\alpha_{i}⊙F_{i}$$

where ⊙ denotes channel-wise multiplication. As defined in Equations (2) and (3), the ACF module adaptively regulates the contribution of different scales, enabling shallow detailed features and deep semantic features to be fused more effectively.

### 4.6. GCA Module

Although the RFE and ACF modules improve local details and multi-scale semantics, distinguishing small lesions from complex backgrounds still requires stronger global reasoning. Because convolutional operations are inherently local, standard architectures often struggle to model long-range dependencies, which limits their ability to suppress background noise. Inspired by GCNet [42] and SCP [43], we designed the GCA module with three parallel branches.

The first branch uses global average pooling (GAP) and global max pooling (GMP) to aggregate global information. The second branch applies a 1 × 1 convolution to generate a linear transformation of the feature map. The third branch also uses a 1 × 1 convolution to produce a simplified query-key interaction.

According to Equations (4)–(6), the GCA module jointly captures channel-wise global context and spatial dependency, thereby improving discrimination between lesion regions and complex non-disease backgrounds.

Let X denote the input feature. The channel-context representation is first obtained as

(4) $$C=\sigma \left(W_{C}GAP\left(X\right)\right)$$

where $W_{c}$ is a learnable transformation and σ(⋅) denotes the activation function. The spatial dependency map is then modeled by

(5) $$S=\sigma \left(Q{\left(X\right)}^{T}K\left(X\right)\right)$$

where Q(⋅) and K(⋅) denote simplified query and key projections, respectively. The final output of the GCA module is expressed as

(6) $$Y=X+\left(W_{v}X\right)⊙C+\left(W_{s}X\right)⊙S$$

where $W_{v}$ and $W_{s}$ denote learnable linear projections.

**Figure 7:**
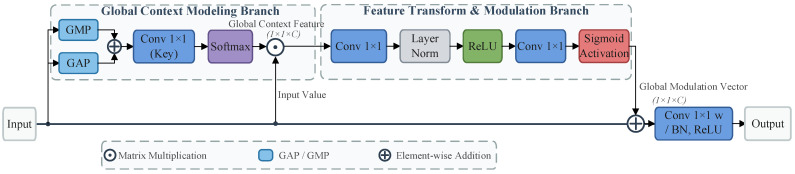
Detailed structure of the Global Context Attention (GCA) module. It captures long-range dependencies by modeling both spatial interactions and channel-wise importance, significantly mitigating non-disease background noise.

The outputs of the first and third branches are then multiplied with the second branch to produce contextual representations across channel and spatial dimensions. Finally, these two contextual branches are combined using a broadcast Hadamard product. Through this design, the GCA module captures both channel-wise and spatial contextual information, thereby strengthening discrimination between lesion regions and complex backgrounds. The structure of the GCA module is shown in Figure 7.

## 5. Conclusions

In this study, we proposed VDD-Net, a feature-enhanced vegetable disease detection network for complex cultivation environments. The framework integrates three key components—the receptive field enhancement module, the adaptive channel fusion module, and the global context attention module—to improve the perception of small lesions, strengthen multi-scale feature fusion, and enhance contextual discrimination under cluttered greenhouse conditions. Experimental results showed that VDD-Net achieved an mAP@0.5 of 95.2% with only 7.78 M parameters on the custom vegetable disease dataset, indicating a favorable balance between accuracy and compactness. In addition, cross-domain evaluation on the PlantDoc dataset demonstrated improved robustness to unseen natural scenes, while edge deployment experiments on Jetson AGX Orin and Jetson Nano confirmed the feasibility of real-time inference. Overall, these findings indicate that VDD-Net provides an effective and deployable solution for intelligent vegetable disease monitoring in modern agriculture. Future work may further extend this framework to video-based disease monitoring, multimodal fusion with environmental sensing data, and broader validation across additional crop species and cultivation conditions.

## Figures and Tables

**Table 1:** Comparison experiments.

Algorithm	mAP@0.5 (%)	mAP@0.5:0.95 (%)	Params/M	FPS
Faster R-CNN	81.8	54.9	41.53	23.9
YOLOv8s	89.0	64.3	11.15	111.4
YOLOv9s	90.3	66.8	7.18	92.6
YOLOv10s	91.1	68.2	7.20	101.5
YOLOv11s	92.5	70.1	9.42	96.3
DETR	81.1	53.1	41.34	34.8
Deformable DETR	85.9	60.5	39.87	42.4
RT-DETR-R18	91.9	69.2	20.00	73.5
Co-DETR	89.3	65.6	80.45	23.3
Ours (VDD-Net)	95.2	76.4	7.78	87.5

**Table 2:** Ablation experiment.

RFE	ACF	GCA	Precision (%)	Recall (%)	mAP@0.5 (%)	mAP@0.5:0.95 (%)	Params/M	FPS
-	-	-	90.1	83.2	91.1	68.2	7.20	101.5
√	-	-	92.1	84.5	93.1	71.0	7.36	95.8
-	√	-	90.8	84.9	92.1	69.8	7.21	100.1
-	-	√	91.5	84.2	92.7	70.6	7.61	89.9
√	√	-	93.2	86.1	94.1	73.5	7.37	93.5
√	-	√	92.8	85.5	93.8	72.9	7.77	87.2
-	√	√	92.4	86.0	93.5	72.2	7.62	89.0
√	√	√	94.4	87.8	95.2	76.4	7.78	87.5

“-”: Indicates that the corresponding module is not included in that experiment. “√”: Indicates that the corresponding module is included in that experiment.

**Table 3:** Comparison experiments for neck network.

Algorithm	mAP@0.5 (%)	mAP@0.5:0.95 (%)	Params/M
BiFPN (without SCF)	93.8	72.9	7.77
BiFPN (SCF-1 = SENet)	94.2	73.8	7.84
BiFPN (SCF-1 = ECANet)	94.0	73.5	7.78
BiFPN (SCF-2)	95.2	76.4	7.78
BiFPN (SCF-3)	94.8	75.7	7.78

**Table 4:** Cross-domain generalization performance comparison on the PlantDoc dataset.

Algorithm	Train Data	Test Data	mAP@0.5 (%)	mAP@0.5:0.95 (%)	Decay (mAP@0.5)
YOLOv10s	PVDD	PVDD	91.1	68.2	-
Ours	PVDD	PVDD	95.2	76.4	-
YOLOv10s	PVDD	PlantDoc	68.3	42.5	↓ 22.8%
Ours	PVDD	PlantDoc	76.5	51.2	↓ 18.7%

“-”: Indicates no performance decay in the given condition. “↓”: Denotes the percentage of performance decay compared to the corresponding model’s performance on the PVDD dataset.

**Table 5:** Comparison of model inference efficiency on edge computing platforms.

Platform	Algorithm	Params (M)	GFLOPs	Latency (ms)	FPS	Power (W)
Jetson Orin	YOLOv10s	7.20	24.5	9.6	104.2	18.4
Jetson Orin	Ours	7.78	26.0	11.2	89.3	19.1
Jetson Nano	YOLOv10s	7.20	24.5	35.1	28.5	7.8
Jetson Nano	Ours	7.78	26.0	41.3	24.2	8.6

## Data Availability

The data presented in this study are available from the corresponding author upon reasonable request.

## Abbreviations

The following abbreviations are used in this manuscript:

VDD-Net	Vegetable Disease Detection Network
RFE	Receptive Field Enhancement
ACF	Adaptive Channel Fusion
GCA	Global Context Attention
mAP	Mean Average Precision
FPS	Frames Per Second
IoT	Internet of Things
PVDD	Protected Vegetable Disease Dataset
